# An Integrated Community Disaster and Climate Resilience Model for rural communities in Zimbabwe

**DOI:** 10.1111/disa.70015

**Published:** 2025-09-18

**Authors:** Louis Nyahunda, Livhuwani David Nemakonde, Sizwile Khoza

**Affiliations:** ^1^ Department of Research and Innovation University of Venda South Africa; ^2^ African Centre for Disaster Studies South Africa; ^3^ Stockholm Environment Institute (Asia Centre) Thailand; ^4^ Social Research Institute Chulalongkorn University Thailand

**Keywords:** climate change, disasters, integration, model, resilience, rural communities, Zimbabwe

## Abstract

This study explores the importance of integrating disaster and climate resilience into Zimbabwe's rural communities, resulting ultimately in a model that reflects both their priorities and needs. The Integrated Community Disaster and Climate Resilience Model (ICDCRM) was developed by engaging with community members, traditional leaders, and disaster risk reduction and climate change practitioners using the constructivist grounded theory approach. A total of 33 participants shared views in focus‐group discussions and semi‐structured interviews—concurrent data collection and analysis were performed with the aid of Atlas.ti (2018) software. This study affirms that disaster and climate resilience strategies can be integrated into at‐risk communities to enhance their responses to climate change and disasters. These phenomena pose interlinked risks; hence, strategies to prepare for and respond to them should be coherent and synergistic. The ICDCRM reveals that integrated disaster and climate resilience can create sustainable pathways to effective coordination of strategies and efficient management of risks.

## 1 INTRODUCTION

Despite a myriad of meanings and conceptualisations in different fields, such as psychology, material science, ecology, and engineering, the resilience concept is a priority for climate change and disaster risk reduction (DRR) agendas (Roostaie, Nawari, and Kibert, 2019). In demonstrating the high regard for resilience on these agendas, the Intergovernmental Panel on Climate Change and the United Nations Office for Disaster Risk Reduction, which are supreme global governing bodies for climate change and adaptation and DRR, respectively, provide comprehensive and similar definitions. Both parties define resilience as the ability of a system to anticipate, absorb, accommodate, or recover from the effects of a hazardous event (Field et al., 2012; UNISDR, 2015). Notably, in DRR and climate change discourse, the use of the resilience concept has led to the emergence of disaster and climate resilience concepts (Manyena, Machingura, and O'Keefe, 2019; Chmutina, Jigyasu, and Okubo, 2020). Like the resilience concept, though, disaster and climate resilience are still riddled with conceptual ambiguities owing to their wide‐ranging application in different disciplines (Djalante et al., 2013; Manyena and Gordon, 2015).

This study finds solace, however, in the definition of Terblanche, de Sousa, and van Niekerk (2022, p. 5), who view disaster resilience as the ability of a community to anticipate, prepare for, and reduce vulnerabilities and risks through increased adaptive and transformative capacities and learning when confronted with hazards and other uncertainties. As for climate resilience, Yadav and Pandey (2024) define it as the ability of a country, region, or community to withstand, address, and recover from related risks, and to progress through continuous social learning and transformation of internal environmental, social, infrastructural, and economic systems. Given the similarities in meanings, there are burgeoning calls for DRR and climate change domains to synthesise and bridge resilience concepts in order to pursue sustainability (Begum et al., 2014; Xu and Kajikawa, 2018).

While disaster and climate change scholars see the imperative need for integrative approaches, frameworks, or models that depict the nexus between disaster and climate resilience still needs to be explored (Roostaie, Nawari, and Kibert, 2019; Xu et al., 2021). A study by Chopde et al. (2024) revealed that there are links between disaster and climate resilience because the concepts focus on the reduction of risk and vulnerability to hazards. The identified ties set the tone for integrating disaster and climate resilience policies, strategies, goals, and programmes; however, there have not been any practical suggestions on how this should unfold. It is also worth noting that debates about integration have centred on pushing governments and policymakers to expedite the integration of disaster and climate resilience (Dias et al., 2019). Furthermore, Nyahunda (2025) posits that countries and communities facing interlinked risks due to disasters and climate change should develop mechanisms for connecting disaster and climate resilience pathways. To date, though, there appear to be no studies with evidence of the implementation of integrated resilience between and across sectors responsible for DRR and climate change adaptation (Nyahunda and Ncube, 2025). This resonates with the inferences of Parker (2020), who posits that organisations responsible for vulnerability and hazard management are yet to recognise the importance of synergies in disaster and climate resilience discourses. What this demonstrates is slow appreciation of the symbiotic relationship between disaster and climate resilience, which, in common practice, hinges on diminishing risks in the human, social, physical, financial, and natural systems (Rana, 2020).

In addition, there is little analysis of the role of local‐level communities in such integration, despite heavy criticism of top‐down approaches in scholarship, because they are bereft of effectiveness and often disregard the diversity of communities and contextual risks (Jamshed et al., 2019). While there are growing calls for integrated disaster and climate resilience, little to no empirical research showcases how local communities can inform this process.

Although there seems to be consensus on the need to integrate disaster and climate resilience, there is a paucity of normative models informed by empirical data in Zimbabwe. This study proposes a novel Integrated Community Disaster and Climate Resilience Model (ICDCRM) that depicts the nexus of the two concepts and is scaffolded by community perspectives. Through the ICDCRM, the study highlights the importance of people‐centred approaches in integration as opposed to relying only on top‐down technocratic expertise, giving little credence to rural communities' knowledge of and capacities in climate change adaptation, disaster management, and overall resilience (Gaillard, Cadag, and Rampengan, 2019). This study is among the earliest of efforts to present community‐driven disaster and climate‐integrated resilience models that foster the development of contextually relevant solutions. The proposed ICDCRM stands to be widely replicated and adapted by various communities in Zimbabwe and beyond for building sustained community resilience that depicts the nexus between disaster and climate resilience, capacities, assets, targets, processes, and outcomes. Practitioners involved in resilience can also use the model when designing and implementing disaster and climate resilience strategies, as it is grounded in community participatory processes.

## 2 THE DISASTER AND CLIMATE RESILIENCE NEXUS: A LITERATURE REVIEW

The relationship between climate change and disasters is vividly clear (Zembe, Nemakonde, and Chipangura, 2022); there is ample scientific consensus that climate change can be depicted as a hazard, source of hazard, or hazard influencer (Fischer and McKee, 2017; Mena et al., 2022). According to Xu et al. (2021), climate change contributes to and exacerbates disaster risks, making it a key source of risks (Kelman, 2015; Hore et al., 2018). Both disaster and climate risks adversely affect the systems on which societies and communities depend (Mena et al., 2022). But these risks are not in isolation, as most disasters experienced by communities are climate‐related. Thus, systems or communities grapple with interlinked disaster and climate risks simultaneously (Xu et al., 2021).

In the wake of climate‐related disasters, effective DRR measures can help build resilience to climate change impacts in the same way as effective climate change management measures can help build resilience to disasters (Kelman, 2015; Mena et al., 2022). The resilience concept is central to DRR and climate change discourses, enabling risk management, hazard exposure reduction, and sustainable development (Kuhlicke, 2013; Fischer and Mckee, 2017).

With similar yet distinct conceptual meanings across climate change and disaster landscapes, resilience is employed in integration philosophies (Hallwright and Handmer, 2021). It is recognised that there is a need to integrate strategies for disaster and climate resilience (Rana, 2020; Xu et al., 2021). Xu et al. (2021) further highlight that integrated approaches to resilience at the community scale can address the fragmented ways at play in relation to institutional, environmental, socioeconomic, and infrastructural systems, inhibiting the attainment of community resilience.

Nonetheless, integrated resilience is still in its infancy in disaster and climate change research and practice (Terblanche, de Sousa, and van Niekerk, 2022). This is despite a broader consensus in the scholarship space about the convergence of disaster and climate resilience capacities (Begum et al., 2014; Tyler, Sadiq, and Noonan, 2019; Rana, 2020). In signposting the nexus between disaster and climate resilience, Gaillard and Jigyasu (2016) infer that both disaster and climate resilience are about capacities. Here, capacities refer to the strengths, assets, and resources that people possess to withstand, cope with, and recover from the disaster and climate hazards that they face (Manyena, 2014; Steiner and Markantoni, 2014). These resources include social networks, technologies, skills, traditional knowledge, human capital, leadership, and institutional, physical, economic, and social means (Kuhlicke, 2013; Manyena, 2014).

It is worth noting that the capacities required for disaster and climate resilience are interconnected and mutually reinforcing (Manyena, Machingura, and O'Keefe, 2019; Terblanche et al., 2022). Critical capacities include absorptive, adaptive, and transformative capacities (Schwanen, 2016). In climate and disaster resilience thinking, absorptive capacity enables systems to absorb change, shocks, or stressors while maintaining their essential functions and structure (Manyena, 2014). Absorptive capacity ignites the abilities of systems to prepare, anticipate, self‐organise, respond, and recover in the face of climate‐related hazards (Fischer and McKee, 2017). Adaptive capacity, meanwhile, is commonly understood as changes or adjustments employed by a system to minimise exposure to perceived and actual hazards or to moderate future damages (Mavhura, 2017). Lastly, transformative capacity is essential to disaster and climate resilience as it is vital for addressing the social construction of disasters, including exposure to climate risks (Mertens, 2017; Manyena, Machingura, and O'Keefe, 2019). Furthermore, Manyena, Machingura, and O'Keefe (2019) state that transformative capacity places communities, individuals, and systems at a vantage point to interrogate their ability to make choices, including the effectiveness of social relations, governance, and structures that constrain their capacities to do so. Hence, transformative capacity challenges the status quo by reconfiguring development structures and risk reduction (Gaillard, Cadag, and Rampengan, 2019).

As the literature suggests, bouncing forward, continuous social learning, and building back better are gaining traction in disaster and climate resilience (Pelling and Manuel‐Navarrete, 2011; Manyena, 2014; Nasi, Jans, and Steg, 2023). The expositions in this section bear testimony to the nexus between disaster and climate resilience, serving as fertile ground for their integration. What requires more analysis, though, is the role of local communities in the integration drive.

## 3 COMMUNITY INVOLVEMENT IN INTEGRATED RESILIENCE

The involvement of local communities in enhancing resilience to disasters and climate change is widely recognised in the literature (Gaillard and Jigyasu, 2016; Jones and Tanner, 2017; Jamshed et al., 2019; Mena et al., 2022). Yet, scholarship has not explored enough the bottom‐up frameworks for integrating disaster and climate resilience. In positing the importance of community involvement in integrated resilience, this article draws from the burgeoning literature on people‐centred approaches to enhancing disaster and climate resilience (Ayeb‐Karlsson et al., 2016; Gaillard, Cadag, and Rampengan, 2019; Mena et al., 2022). The importance of people‐centred or bottom‐up approaches in resilience stems from the need to challenge the reliance on technocratic and top‐down approaches to enhancing community resilience (Oxley, 2013; Wisner, Gaillard, and Kelman, 2015). As Gaillard, Cadag, and Rampengan (2019) argue, risk reduction in resilience thinking relies on technocratic strategies involving the transfer of knowledge, experience, and technology. However, there is a broader consensus in the literature regarding local communities' abilities to deal with climate‐related and other natural hazards on their own (Gaillard and Jigyasu, 2016; Jones and Tanner, 2017; Jamshed et al., 2019; Nasi, Jans, and Steg, 2023).

The recognition that local communities have capacities underpins the imperative to involve them in designing and implementing integrated disaster and climate resilience strategies that work in their contexts (Oxley, 2013). Through people‐centred integrated resilience, communities understand their interlinked risks and devise synchronised and complementary measures to mitigate them (Imani, Fakour, and Lo, 2021). This means that communities should identify the interlinkages between the risks posed by climate change and disasters, and the integrated response measures should be people‐centred (Chopde et al., 2024). As Mutiarni, Nakamura, and Bhattacharya (2022) note, the integration of disaster and climate resilience should be geared towards fostering the participation of at‐risk communities. This is because local communities always constitute the first line of defence in building resilience and reducing vulnerabilities (Manyena, 2016; Mutiarni, Nakamura, and Bhattacharya, 2022).

Furthermore, communities affected by disasters and climate‐related hazards are resourceful and knowledgeable (Gaillard, Cadag, and Rampengan, 2019; Imperiale and Vanclay, 2021). This study infers that integrating disaster and climate resilience is the prime responsibility of those at risk; they should occupy the vanguard in designing their context‐specific strategies informed by everyday and long‐term priorities. In corroboration, Mutiarni, Nakamura, and Bhattacharya (2022) opine that community‐based integrated approaches can foster the development of culturally appropriate, locally relevant, and resource‐efficient solutions. By relying on community‐based approaches for integrating disaster and climate resilience strategies, this study argues that outsiders should not control risk perception and management in disaster and climate resilience, as this may render at‐risk communities helpless. Furthermore, top‐down and technocratic actions should not replace community capacities and innovations but rather support local people's aspirations according to the community's terms.

## 4 STUDY SETTING

Chimanimani district in the Manicaland province of Zimbabwe was the case study for proposing the ICDCRM. Its selection was based on evidence in the literature, which suggests that the district has a high climate change‐induced disaster risk profile (Chanza et al., 2020; Nyahunda, Tirivangasi, and Mabila, 2022; Tirivangasi and Nyahunda, 2025b). In Chimanimani, there are several indicators of climate change, characterised by water stress, drought, pasture depletion, increased dry spells, declining or extreme rainfall, and disease epidemics (Chanza et al., 2020). Chimanimani is also susceptible to hazards such as floods, cyclones, and landslides (Mutandwa, Hanyani‐Mlambo, and Manzvera, 2019; Venganai and Mupoperi, 2023). In addition, climate change manifestations interact with other risk drivers, including poverty and unemployment, to perpetuate the vulnerability of communities (Tirivangasi and Nyahunda, 2025a). In the face of these compounding problems, the evidence in the literature also suggests that communities in Chimanimani district are not passive victims of disasters and climate change; instead, they respond to such shocks and stressors with a cocktail of strategies, either collectively or individually (Marango and Chitongo, 2021).

The need to rely on rural communities' perspectives, experiences, and aspirations in Chimanimani district was harnessed by the study to propose an integrated model that depicts the complementarities between disaster and climate resilience. As such, the ICDCRM was developed by engaging with community members, traditional leaders, and DRR and climate change practitioners. Study participants were drawn from seven wards in the district: Bvumbura; Chikukwa; Chimanimani; Nechirinda; Ngorima; Nhedziwa; and Tiya.

## 5 METHODOLOGY

This study required a methodology that would enable the development of a model informed by empirical data from the field. Consequently, the constructivist grounded theory (CGT) approach was adopted. Charmaz (2017a) defines CGT as an inductive methodology that builds models from empirical data. As such, CGT provides procedures and guidelines for collecting and analysing data to generate a conceptual model that reflects the lived experiences of the participants (Mitchell, Jr., 2014; Charmaz, 2020). By selecting the CGT approach, the study envisaged a model that reveals the participants' views, perceptions, and experiences (Mohajan and Mohajan, 2022). Charmaz (2017b) posits that CGT fosters the development of qualitative traditions by studying the experiences of those who live them. The approach allowed the researchers to explore the need for an integrated disaster and climate resilience model from the standpoint of the participants (Charmaz, 2020). We gathered diverse perspectives on and insights into the necessity of integrating disaster and climate resilience by considering the social dynamics of the communities that can contribute to an ICDCRM.

### 5.1 Sampling, sample size, data collection, and analysis

In grounded theory studies, purposive sampling is used to identify the first cohort of participants for the first wave of data collection and analysis (Charmaz, 2020). Creswell (2017) postulates that purposive sampling allows for the selection of participants according to the study's needs, aiming to expose the phenomena under investigation. After the first round of data collection, the theoretical sampling technique was employed, whereby the themes that emerged initially determined the questions asked, and the selection of individuals deemed appropriate to answer them, in subsequent rounds (Kenny and Fourie, 2015). The key questions asked during data collection related to whether communities see a need to integrate disaster and climate resilience; other questions pertained to suggestions regarding an ICDCRM. Beyond the emerging themes connected to these questions, additional participants were chosen based on the need to have a variety of perspectives, since the model required a diverse representation of community aspirations. CGT's concurrent data collection and analysis features determined the study's sample (Mohajan and Mohajan, 2022). Data were gathered through focus‐group discussions (FGDs) and semi‐structured interviews (SSIs). FGDs involved community members, whereas SSIs involved traditional leaders and DRR and climate change practitioners.

As data were collected from a particular cohort of participants, they were analysed concomitantly. The themes and categories that emerged in data analysis informed the discussions with another set of participants. This process led to the study sample being established in three phases through theoretical sampling. Charmaz (2020) postulates that theoretical sampling depends on pursuing an iterative process and thoroughly checking the constructed categories against the data. Initial coding and focused coding were used to develop the categories leading to production of the model. Atlas.ti (2018) software was used to organise the patterns that emerged from the categories.

That being the case, the first phase entailed one FGD and two SSIs, one with a traditional leader and one with a DRR practitioner. FGD participants were selected from one community, whereas the practitioners were selected from various locations in Chimanimani district. The traditional leaders were selected from the respective communities where FGDs were conducted. Each FGD had five participants. The second phase had two FGDs conducted in different communities; the first one had seven participants, and the second one had six participants. This phase also involved SSIs with two traditional leaders and one climate change practitioner.

After this round of data collection and analysis, the researchers developed the draft model based on the data from the field. The tentative ICDCRM was shared with another cohort of participants to see if new information emerged and to allow them to comment on the model. This third wave encompassed one FGD with six participants and SSIs with two practitioners and two traditional leaders. This was done to establish theoretical sampling and theoretical saturation (Charmaz, 2017a). The new information that emerged during the third phase was incorporated in the final model. Theoretical saturation was confirmed empirically as no new insights were emerging from the data. A total of 33 participants shared their views on the study.

## 6 FINDINGS

This section presents the findings that inform the elements of the proposed ICDCRM. The themes that emerged from the data also inform the subthemes that will be discussed in conjunction with the description of the model.

### 6.1 Perceptions about hazards faced by communities

The study solicited information about the hazards faced by communities in Chimanimani district, Zimbabwe. This information was deemed crucial in ascertaining their knowledge of the hazards they face, how they impact them, and the measures to be taken to mitigate such dangers. The hazards faced by communities in Chimanimani encompass, inter alia, drought, floods, strong winds, wildfires, soil erosion, death of livestock, water shortages, storms, heatwaves, extremely cold temperatures, rock falls, disease epidemics and pandemics, and water conflicts between households and domestic animals. The following narrations represent some of the views shared by the participants:

*The main challenge is drought. Even if we look at what is happening in the fields now, it is all drought, so that is the main problem. Water shortage is another challenge. We now compete with domestic animals for water* (FGD).

*Another challenge we face with our livestock is tick‐borne diseases, especially during the rainy season* (SSI).

What also emerged from the participants is that these hazards pose serious risks to their lives and livelihoods. Hence, they employ a raft of measures to deal with them, including livelihood diversification, the use of indigenous knowledge, and support systems of government and non‐governmental organisations (NGOs) that provide resilient livelihood and other empowerment projects such as apiculture, horticulture, and sustainable farming practices. The government and NGOs also embark on public awareness of hazards, risk analysis, and early warning dissemination.

### 6.2 Views on integrated disaster and climate resilience

Community members' and practitioners' views on integrating disaster and climate resilience were identified. This information was deemed to be an integral part of developing the ICDCRM. Since the participants identified the hazards they faced, exploring their views on integrative resilience was valuable. The study established that communities faced interlinked hazards, most exacerbated by climate change. More importantly, the communities that are adversely affected by these hazards do not distinguish between whether or not the hazards are related to climate change. As a result, there was a consensus that disaster and climate resilience serve the same purpose and need to be integrated. The participants had the following to say:

*It is important to integrate the two to save limited resources* (FGD).

*Considering that climate change triggers many disaster risks, communities now face these risks as a package and not in isolation. It, therefore, defies logic to treat resilience approaches to disasters and climate change as discrete because they are the same* (SSI).

What can be underscored here is that climate change‐related disaster risks pose similar threats to communities. Thus, response measures need to demonstrate some synergy and interdependence. The simultaneous manifestation of diverse hazards, most of which are climate‐related, was believed to be among the key reasons for integrating disaster and climate resilience. Practitioners emphasised that the integration should start at the policy level to enable departments and various institutions to co‐exist and synergise their approaches in order to stimulate the impact of their services.

### 6.3 Community needs to be addressed for disaster and climate resilience

After the study established that most participants identified a need to integrate disaster and climate resilience, they were asked to indicate the resources or measures required to build resilience to related events. They stressed that periodic awareness of hazards and resilience building should capture how hazards and resilience are conceptualised in local terms. These are understood as: hazards (*njodz*i); floods (*mafashamu*); famine (*nzara*); tropical storm (*dutu mupengo*); heatwaves (*mudziyira*); wildfires (*moto wesango*); gullies (*makoronga*); disease epidemics or pandemics (*zvirwere/hosha*); and soil erosion (*kukurwa kwevhu*). Other community needs included: community mobilised funds; evacuation centres; functional local institutions; employment creation; poverty reduction; periodic awareness of hazards and resilience in local languages; access to technology; and healthcare facilities. A comprehensive description of needs was mentioned across all study sites. In most cases, similar needs kept recurring in every FGD and SSI. For instance:

*The local structures we have are not that effective because of resource constraints. As it stands, they need to be capacitated to coordinate our activities effectively. They must be functional* (FGD).

*When the houses have been destroyed, it takes forever to be relocated, and the struggle continues. We need evacuation centres to be built in every community. In the previous disaster, we had to use churches as evacuation centres, and they could not accommodate everyone* (FGD).

The highlighted needs represent what communities aspire to have in terms of building resilience to disasters and climate risks.

### 6.4 Community assets for disaster and climate resilience

The study also established that rural communities have an array of assets that can be utilised in building disaster and climate resilience, including a sense of collective responsibility, unity, and mutual support hinged on social networks, further fostering collective decision‐making when addressing community problems. It can be posited that collective efficacy is invaluable in cultivating community resilience. The findings revealed that collective efficacy, the support systems of development agencies and government, and the availability of natural, cultural, human, and physical resources are essential assets that can be employed in integrated resilience building. Furthermore, the study found that livelihood diversification, indigenous knowledge systems, availability of local leadership, and the community's knowledge of resilience also serve as critical determinants of resilience when pursuing integrated approaches. Participants pointed out:

*Yes, we have the knowledge. As the local leadership, we play a very crucial role. We always see the results when we perform rituals for [Doro Remakoto] rainmaking. The knowledge we have is important* (FGD).

*When we talk about resilience, we cannot downplay the importance of human, natural, and physical resources available in these communities. These resources are critical for building resilience* (SSI).

From the findings, we can assert that communities can rely on the above assets to foster community resilience.

### 6.5 People‐centred strategies for integrated disaster and climate resilience

After identifying the links between disaster and climate resilience and the need for integrated resilience, the participants were asked to suggest what integrated strategies for disaster and climate resilience would look like for communities and practitioners. The common thread in the responses was that they must be people‐centred, and local communities must be seen as partners with whom the integrated agenda is achieved. The participants suggested strategies such as knowledge integration (scientific and indigenous), inclusive hazard mapping, resilient livelihoods and strong social safety nets, continuous social learning, capacity building and education, community‐based monitoring and evaluation, and sectoral alignment of land use, water, energy, and agriculture policies. One participant stated:

*In my view, the identification of hazards should involve community members. Now we see strangers being involved in locating households or locations prone to hazards as if they know our area more than us* (FGD).

In the light of the suggested strategies, the study classified them as socioeconomic, institutional, infrastructural, and environmental strategies for disaster and climate resilience.

## 7 DISCUSSION

The findings of this study, as presented in the section above and the literature consulted, provided sufficient corroboration of the importance of and the need to integrate efforts to build disaster and climate resilience. Yet, most of the literature consulted does not provide mechanisms for doing so. To fill this gap, this paper proposes a normative ICDCRM, which endeavours to showcase the symbiotic relationship between the disaster and climate resilience concepts. This is informed by their complementary capacities and strategies, which communities and practitioners have to follow. The novel model represents a starting point for building integrated community resilience to disasters and climate risks.

### 7.1 Target users of the ICDCRM

The proposed model targets rural communities grappling with diverse climate‐related disaster risks and striving to build resilience against them. These communities and the agencies supporting them can use the model to enhance their preparedness, timely recovery, and risk reduction and management, and they can bounce forward when faced with disaster and climate risks. The ICDCRM provides mutually reinforcing elements that can augment community resilience. Government departments and development agencies can rely on the elements of the model to acknowledge synergy of their strategies. This would be a step forward in pushing the integration of disaster and climate resilience at the national level, cascading down to communities. Integrated approaches can assist such actors with reimagining their interventions.

## 8 DESCRIPTION OF THE ICDCRM

**FIGURE 1:**
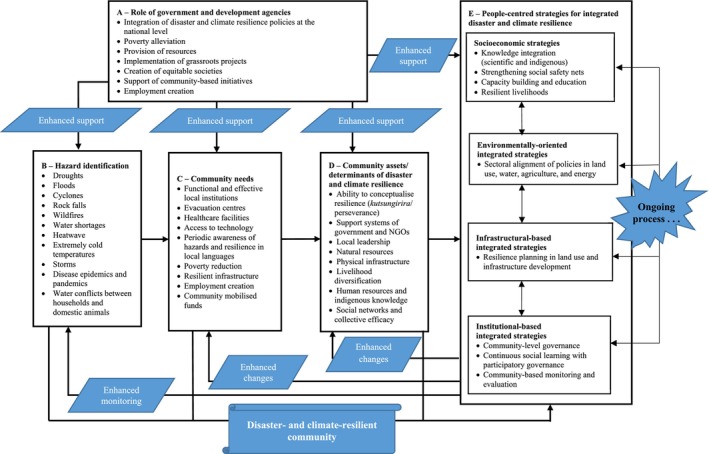
The ICDCRM. **Source:** authors.

The proposed model comprises five interconnected components, as shown by the black arrows in Figure 1: (i) role of government and development agencies; (ii) hazard identification; (iii) community needs; (iv) community assets/determinants of disaster and climate resilience; and (v) people‐centred strategies for integrated disaster and climate resilience. The model is to be read from top to bottom (A to B) and then from left to right (B to E). The following subsections provide detailed discussions of the components of the ICDCRM.

### 8.1 Role of government and development agencies

Component A of the model represents the role of government and development agencies in building and supporting integrated disaster and climate resilience. As illustrated, rural communities cannot overcome the hazards they face or thrive in their resilience building endeavours single‐handedly; service providers such as the government and NGOs are vital in supporting community initiatives and reducing vulnerabilities. Initially, the government must make efforts to integrate disaster and climate resilience at the policy level. The government and development agencies can use this model to establish the merits of integrating resilience building goals at the national level. It is worth noting that the ICDCRM complements their existing efforts, although these need to showcase the synergy and complementarities between disaster and climate resilience goals, programmes, and strategies. As depicted in the blue boxes inscribed ‘enhanced support’ in Figure 1, the government and NGOs play significant roles in supporting other components such as hazard identification, meeting community needs, supporting community assets for resilience, and enhancing people‐centred strategies for integrated disaster and climate resilience.

### 8.2 Hazard identification

Component B of the model depicts the hazards prevalent in Chimanimani district. The findings of the study revealed a myriad of hazards prevalent in the study area, including drought, floods, cyclones, storms, high and low‐temperature extremes, strong winds, wildfires, and water resource conflicts. The communities also face hazards in the form of disease epidemics and pandemics, pests, tick‐borne diseases, gullies, soil erosion, and rock falls. By identifying them, the study infers that participants demonstrated awareness and understanding of their challenges. With knowledge of the hazards within their vicinity, communities can take proactive measures and formulate solutions to address them, pinpointing relevant resources and service providers. This is in line with assertions by scholars such as Agrawal, Elliott, and Simonovic (2020) who posit that the ability to identify hazards stimulates strategies to mitigate them and assists with proactive decision‐making.

### 8.3 Community needs

Component C of the model lists the services and resources that participants said they lacked or needed to enhance their resilience strategies. The study established that the needs that must be met for communities to be resilient include local institutions that are functional and effective, evacuation centres, healthcare facilities, poverty reduction, resilient infrastructure, and employment creation. These needs are connected to the role of government because it shoulders the primary responsibility for ensuring that these requirements are fulfilled. In the same vein, development agencies complement the role of government in providing services aimed at meeting community needs. Collectively, these institutions offer enhanced support to communities through poverty alleviation mechanisms and the implementation of grassroots projects. With regard to functional and effective local institutions, both governmental and non‐governmental actors are privy to the lived realities of their communities and can facilitate resilience strategies that resonate with their priorities. It is important that the roles that these bodies play within communities are highly recognised, and resources should be channelled towards capacitating them, as they are the first point of contact for communities seeking help during emergencies.

When these institutions are capacitated and coordinate their efforts, disaster and climate resilience can be easily realised, because they would shoulder responsibility for sharing information within communities. Drawing on Obi and Babatunde (2019), this paper argues that these institutions can also be avenues for downward accountability, in contrast to top‐down approaches, where local communities cannot hold external service providers to account. Evacuation centres, healthcare facilities, and access to technology represent some of the community's needs that must be addressed in pursuit of disaster and climate resilience. From the findings, a general view emerged that there are still no evacuation centres that can serve as safe havens for disaster survivors, despite the ravaging of communities by past disasters. Those in power must make provision for these centres. Additionally, the ICDCRM proposes that resilience thinking should be applied in constructing resilient infrastructure that can be used as evacuation centres in the wake of hydrometeorological hazards such as floods and cyclones. This is meant to reduce the casualties that typically occur when displaced people have nowhere to go. Lastly, access to technology is essential for risk assessment, including hazard mapping, remote monitoring of hazard‐prone areas, the implementation of early warning systems, communication during emergencies, and the facilitation of education and awareness. All of these aid the fostering of community resilience (Nasi, Jans, and Steg, 2023).

The findings also revealed that communities need locally mobilised funds that can be tapped in times of emergency. This model asserts that most integrated strategies require adequate funding, and communities in Chimanimani district cannot rely solely on the government, which does not have clear funding mechanisms for DRR and resilience (Mavhura, 2021). Locally generated funds for resilience building have been lauded as measures to break away from a cycle of dependency on external help, which is riddled with corruption and a lack of accountability. Given periodic awareness of hazards and resilience in local languages, the ICDCRM infers that community resilience is a function of the comprehensibility of information that resonates with local people's cultural dynamics and level of understanding.

The results showed that local languages are not considered in resilience framing and hazard awareness. This means that resilience programmes championed by various developmental agencies in Chimanimani are predominantly framed in English, not in local languages (such as *Shona*). We argue that the exposition that resilience discourse lacks local languages or what the term means in diverse languages is uncontested (Nyahunda, Nemakonde, and Khoza, 2024). Thus, the proposed model suggests that integrated resilience strategies should be grounded in a local conceptualisation of various terms that refer to hazards and the response measures to be undertaken. We contend that mainstreaming periodic hazard awareness and resilience in local languages can dismantle the pervasive neoliberal and Western ideologies concerning resilience.

Poverty reduction and employment creation emerged as among the community needs to be addressed for disaster and climate resilience. This is because poverty levels and unemployment are rampant in Zimbabwe, making most individuals and communities vulnerable to hazards. It was revealed that the poverty traps in which most households find themselves hinder communities from preparing for, coping with, and surviving disasters (resilience). It can be postulated therefore that building disaster and climate resilience depends on the reduction of poverty levels and employment creation, which should enable households and individuals to deal with hazards on their own instead of relying on external help alone.

### 8.4 Community assets or determinants of disaster and climate resilience

Component D of the model portrays the assets that can be utilised by communities to address their needs and inform the strategies to be employed to build disaster and climate resilience. As illustrated, assets that inform rural communities' capacities to develop resilience strategies include their ability to conceptualise resilience (*kutsungirira*/perseverance), support systems of government and NGOs, local leadership, natural resources, physical infrastructure, livelihood diversification, human resources and indigenous knowledge, and social networks and collective efficacy. These form the core of determinants of resilience building. The ability to conceptualise resilience sets the tone for how communities can innovate and apply diverse knowledge to the building of resilience. The support systems of government and NGOs stimulate local‐level action and provide resources that can assist communities in preparing for disasters or recovering after experiencing shocks.

Furthermore, local leadership plays a vital part in communicating information and coordinating resources, as well as in coordinating disaster and climate resilience strategies. Traditional leaders are on the frontlines in terms of steering the direction of how communities prepare for and respond to disaster and climate change threats through resilience building. In addition, local leadership is also a vehicle for downward accountability and monitoring resilience strategies in communities.

The availability of diverse human capital, such as community leaders, educators, small business owners, volunteers, farmers, youths, and the elderly, means that Chimanimani district has a strong resource base that can devise and bring together the diverse expertise required to respond to the various hazards confronted.

The availability of human resources is complemented by natural resources such as water sources, agricultural land, forests, and timber plantations. In the context of this model, the availability of natural and human resources means communities can innovate and acquire the knowledge and skills necessary for preparedness and bouncing forward in the face of uncertainties and shocks. Collective efficacy and social networks are intangible and important components when building disaster and climate resilience. It can be postulated, therefore, that building resilience hinges on the level of cooperation, mutual understanding, information sharing, shared responsibilities, and interactions among individuals across communities. This gives credence to Aldunce et al. (2014) who opine that communities with more robust networks, trust, and civic engagement demonstrate strong social cohesion and collective action to enhance community resilience in the face of hazards and disturbances. Lastly, livelihood diversification enables communities to have diverse income portfolios and means of living, which is a poverty reduction mechanism and a vehicle with which communities can acquire other material assets essential for resilience. Rural communities also rely on indigenous knowledge, which is important for giving direction on land use practices, water and disaster management, and climate change adaptation. All of these assist with the building of disaster and climate resilience.

### 8.5 People‐centred strategies for integrated disaster and climate resilience

Component E of the model represents people‐centred strategies to integrate disaster and climate resilience. The ICDCRM underscores that such strategies are the cornerstones of community resilience. First, they ensure that communities are actively involved in designing, implementing, analysing, monitoring, and evaluating disaster and climate resilience initiatives or strategies. Second, they may address the fragmentation of disaster and climate resilience concepts in policy and practice. The findings of this study reveal that disaster and climate risks disrupt the functioning of communities in the socioeconomic, institutional, environmental, and infrastructural spheres. Consequently, at‐risk communities need to devise related strategies to enhance disaster and climate resilience. Socioeconomic strategies, for instance, involve inclusive hazard mapping, risk management and reduction, strengthening social protection systems, and pursuing resilient livelihoods. And through people‐centred institutional strategies, institutions supporting communities with resilience‐building efforts must engage in the sectoral alignment of policies, plans, and interventions.

The blue boxes in Figure 1, labelled ‘enhanced changes’, mean that the implementation of people‐centred strategies to integrate disaster and climate resilience would lead to changes in community needs. Thus, they would allow communities to address their needs and foster changes in how they manage their assets and monitor diverse hazards, as depicted by the ‘enhanced monitoring’ blue box.

The ICDCRM portrays an ongoing process that can be measured as outcomes. For example, hazard mapping, continuous social learning, risk reduction and management, capacity building, participatory governance, periodic awareness of hazards and resilience, pursuing resilient livelihoods, strengthening social safety nets, and use of indigenous and scientific knowledge are deemed to be ongoing processes, with the outcome being a disaster‐ and climate‐resilient community. The ongoing process of developing resilience strategies accommodates changes in the spatial and temporal characteristics of hazards.

## 9 CONCLUDING REMARKS

This study suggests a normative model to integrate disaster and climate resilience; it is a product of the aspirations, viewpoints, and beliefs of communities and practitioners in Chimanimani district, Zimbabwe, and the literature consulted. The ICDCRM is among the earliest efforts to signpost the nexus between disaster and climate resilience and efforts to integrate them.

The model is unique in that it is tailor‐made for the context‐specific risks and resilience aspirations of Chimanimani district. The novelty means that it has not been tested in a real‐life situation. Another limitation is that it is context‐specific, which may impede its generalisability. Yet, this research is a good starting point to guide efforts to build disaster and climate resilience in an integrated manner.

We further submit that the ICDCRM needs to be tested in practice and adapted for communities other than those where the study was conducted. The proposed model recommends that integrated disaster and climate resilience ambitions in policy and practice need to be people‐centred because local people directly face disaster and climate risks and are critical knowledge bearers and partners in integrated resilience. We strongly encourage policymakers, government agencies, and community stakeholders to adopt integrated resilience strategies to foster efficient, coherent, and effective pathways for addressing disaster and climate change risks.

The ICDCRM stands a good chance of being adapted to other regions facing disaster and climate change risks. The departure point here is to acknowledge the importance of coherent and synergistic strategies in tackling disaster‐ and climate change‐related vulnerabilities and risks. This should be followed by the development of integrated resilience strategies that are responsive to diverse community needs, beliefs, and aspirations.

## CONFLICT OF INTEREST STATEMENT

There are no competing conflicts of interest to report in the writing and publishing of this article.

## Data Availability

The data that support the findings of this study are available on request from the corresponding author. The data are not publicly available due to privacy or ethical restrictions.
